# The Expression of Toll-Like Receptors in Patients with B-Cell Chronic Lymphocytic Leukemia

**DOI:** 10.1007/s00005-016-0433-7

**Published:** 2017-01-12

**Authors:** Justyna Rybka, Aleksandra Butrym, Tomasz Wróbel, Bożena Jaźwiec, Aleksandra Bogucka-Fedorczuk, Rafał Poręba, Kazimierz Kuliczkowski

**Affiliations:** 1 Department of Hematology, Blood Neoplasms and Bone Marrow Transplantation, Wroclaw Medical University, Pasteura 4, 50-367 Wroclaw, Poland; 2 Department of Physiology, Wroclaw Medical University, Wroclaw, Poland; 3 Department of Internal, Occupational Diseases, Hypertension and Clinical Oncology, Wroclaw Medical University, Wroclaw, Poland

**Keywords:** Toll-like receptors, Chronic lymphocytic leukemia

## Abstract

B-cell chronic lymphocytic leukemia (B-CLL) presents with progressive accumulation of monoclonal B cells in the peripheral blood, bone marrow and lymphoid organs. B-CLL is characterized by heterogeneous clinical outcome. The expression of Toll-like receptors (TLRs) and their association with other prognostic factors in B-CLL patients remain unclear. The aim of our study was to evaluate the expression of TLR2, TLR4 and TLR9 genes and their significance as biological markers in patients with B-CLL. Sixty patients with newly diagnosed B-CLL were evaluated. The healthy control group included 20 age-matched individuals. Using quantitative reverse transcriptase PCR, the mRNA expression of genes TLR2, TLR4 and TLR9 was measured. TLR4 gene expression was lower in B-CLL patients as compared to the control group and TLR2 gene expression was higher in B-CLL patients than in healthy individuals. TLR9 gene expression was higher in the control group than in patients with B-CLL. TLR4 mRNA expression was lower in patients with advanced-stage CLL (Rai stages III and IV) than in patients with early stage disease (Rai stages 0–II). TLR2 gene expression was higher in patients with advanced-stage CLL (Rai stages III and IV) than in patients with early stage disease (Rai stages 0–II; *p* < 0.05). Our results suggest that TLRs could become potential biological markers for the clinical outcome in patients with B-CLL.

## Introduction

B-cell chronic lymphocytic leukemia (B-CLL) is the most common proliferative hematological disease in Western Europe and in the United States. B-CLL is associated with clonal proliferation of abnormal B lymphocytes, present in peripheral blood, bone marrow and lymphoid and extranodal organs (Chiorazzi et al. 2005). The pathogenesis of CLL is multifactorial and is associated with the activation of immune processes and with abnormal apoptosis (Caligaris-Cappio 2000). B-CLL has a variable clinical course and presentation. In some patients, the disease is chronic and does not require treatment for a long time, while in other patients the course of B-CLL is very aggressive. The negative prognostic factors in B-CLL include expression of ZAP70 and CD38, cytogenetic irregularities (deletion of 17p and/or 11q), and absence of somatic mutations in the immunoglobulin heavy chain variable (IGHV) genes (Zenz et al. 2010).

Toll-like receptors (TLRs) are a crucial element of innate immunity. Up to date, 11 TLRs have been identified in the human body (TLR1-11) (Akira and Takeda 2004). TLRs appear on the surface of lymphocytes, monocytes, macrophages, and granulocytes. Through contact with the corresponding ligands, TLRs initiate a series of immune reactions which determine an anti-inflammatory response (Kawai and Akira 2007). TLRs are involved in the active processes connected with sepsis, autoimmune diseases, cancer as well as liver and cardiovascular diseases (Kawai and Akira 2011). Expression of TLRs on the surface of tumor cells may indicate their role in carcinogenesis and in their influence on tumor development and growth. Promotion of cancer is often proceeded by chronic inflammation which can also testify to the involvement of TLRs in malignant clonal expansion (Balkwill and Coussens 2004). It was also found that TLRs are involved in transferring cell signals through MAPK and PI3K cascades whose individual elements become disrupted during carcinogenesis (Hua and Hou 2013). The stimulation of TLRs plays a crucial role in the homeostasis of mature B cells; however, their participation in the etiology of CLL remains unknown. TLR2, TLR4 and TLR9 are involved in proliferation and differentiation of B lymphocytes.

The aim of our study has been to evaluate expression of TLR2, TLR4 and TLR9, and their relevance as prognostic factors in patients with B-CLL.

## Materials and Methods

This study included 60 patients with B-CLL (29 women and 31 men). The median age of the patients was 68 (range 52–88). The examination protocol was approved by the Bioethical Commission. The diagnosis of B-CLL was consistent with the criteria IWCLL/NCI for CLL. 40 patients (67%) were in the early stages of clinical progression according to the Rai staging system (0–II) and 20 patients (33%) were in stage III or IV according to the Rai staging system. All patients were previously untreated. The control group consisted of 20 healthy individuals (10 women and 10 men) in the age range similar to the studied population. Clinical data of the patients are shown in Table 1.

**Table 1:** Clinical data of patients

Number of patients	60
Age	68 (range 52–88)
Median of hemoglobin level (g/dl)	13.3 (range 6.3–17)
Median of platelets count (×10^6^)	181 (range 60–303)
Median of white blood cells level (×10^6^)	27.59 (range 12.0–365.0)
Median of lymphocytes percentage	78.5 (range 59–94)
Median of beta2-microglobulin level (mg/l)	3.0 (range 1.3–4.9)
Median of lactate dehydrogenase level (U/l)	391 (range 240–1199)
Cytogenetics unfavorable	5 patients
ZAP-70-positive	14 patients
CD38-positive	18 patients
Stage of CLL (Rai staging system)	0–II: 40 patients III–IV: 20 patients

Mononuclear cells of CLL patients and age-matched healthy donors were obtained from peripheral blood by density gradient centrifugation (Gradisol L, AquaMed, Łódź) and stored at −75 °C in RNA isolation reagent (TriReagent Solution, Ambion/ThermoFisher) or as dry pellet.

Total RNA was extracted from frozen cells by modified Chomczynski method according to the instruction delivered with TriReagent Solution and reverse-transcribed to cDNA with RNA-to-cDNA kit (ThermoFisher) (Chomczynski and Sacchi 1987).

Relative expression of TLRs was assessed by real-time PCR using inventoried TaqMan^®^ Assays from Life Technologies/ThermoFisher: Hs01152932_m1 for TLR2, Hs0015299_m1 for TLR4 and Hs0015973_m1 for TLR9. Beta-glucuronidase (GUSB) served as endogenous control (Hs99999908_m1). Reaction was performed in 7500 Real-Time PCR instrument (LifeTechnologies), using Gene Expression MasterMix (LifeTechnologies/ThermoFisher). Comparative C_T_ method was used to compare the expression among patients and with healthy controls (Schmittgen and Livak 2008).

The results were statistically analyzed using STATISTICA 8.0. Statistical analysis was performed by means of Mann–Whitney *U* test and *p* < 0.05 indicated a significant difference. Progression-free survival (PFS) was determined using Kaplan–Meier method. The long-rank test was used to compare the curves.

## Results

### TLRs Expression in Control Group

In comparison to control group, TLR2 gene expression was higher in B-CLL patients than in healthy individuals (ΔCt TLR2 6.46 ± 9.58 vs 0.98 ± 0.43; *p* < 0.05). TLR4 gene expression was decreased in B-CLL patients in comparison to control group (ΔCt TLR4 2.21 ± 0.32 vs 11.91 ± 70.22; *p* < 0.05). TLR9 gene expression was higher in the control group than in patients with B-CLL (ΔCt TLR9 23.65 ± 16.29 vs 3.35 ± 1.93; *p* < 0.05).

### TLRs Expression and Stage of CLL

TLR2 gene expression was higher in patients with advanced-stage CLL (Rai stages III and IV) than in patients with early stage disease (Rai stages 0–II; *p* < 0.05). TLR4 gene expression was lower in advanced disease than in early stages (*p* < 0.05). The results are shown in Table 2.

**Table 2:** Correlation between mRNA expression of TLRs and stage of disease

	Advances stages (*x* ± SD) (*n* = 20)	Early stages (*x* ± SD) (*n* = 40)	*p*
ΔCt TLR2	19.10 ± 17.76	7.94 ± 6.25	<0.05
ΔCt TLR4	27.88 ± 75.18	53.10 ± 63.49	<0.05
ΔCt TLR9	3.98 ± 1.85	3.26 ± 2.55	ns

*n* number of patients, *x* mean, *ns* not significant, *SD* standard deviation

### TLRs Expression and Clinical Characteristics of Patients

We observed that expression of TLR2 in patients with anemia was significantly higher than in patients without anemia (ΔCt TLR2 17.18 ± 14.29 vs 7.10 ± 5.98; *p* < 0.05).

We found significant positive relationships between TLR2 and white blood cells (*r* = 0.50, *p* 0.05), TLR2 and lactate dehydrogenase (*r* = 0.36, *p* < 0.05), TLR2 and beta2 microglobulin (*r* = 0.33, *p* < 0.05) and TLR4 and white blood cells (*r* = 0.29, *p* < 0.05). In addition, there was a statistically significant negative relationship between TLR2 and hemoglobin level (*r* = –0.43, *p* < 0.05).

### TLRs Expression and ZAP70 and CD38 Expression

We observed that in patients with positive ZAP70 expression, TLR4 expression was lower than in patients with negative expression (ΔCt TLR4 3.25 ± 0.52 vs 9.95 ± 3.22). This result was not confirmed by statistical analysis due to a limited number of cases. We showed no association between TLRs expression and CD38 expression.

### Progression-Free Survival

**Fig. 1:**
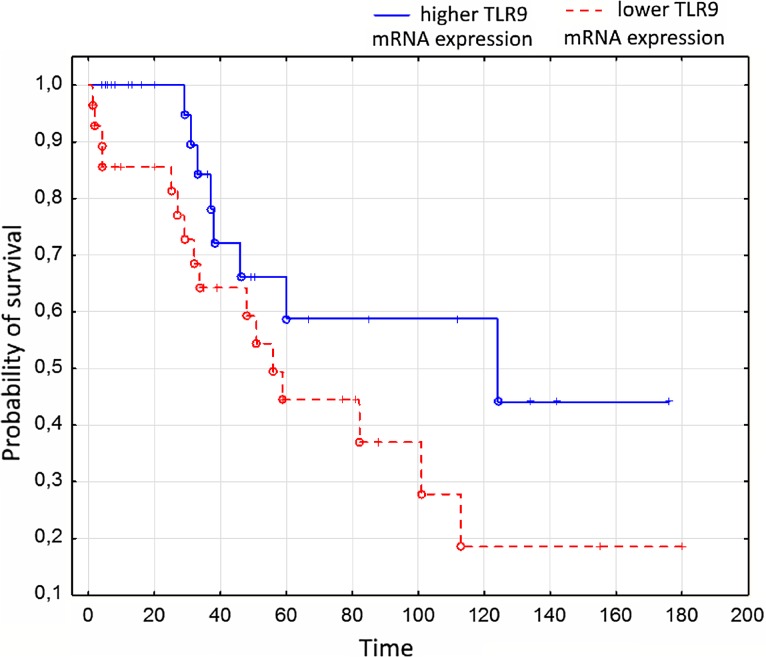
The Kaplan–Meier estimate of PFS in patients with CLL and different TLR9 expression

The median follow-up was 48 months (range 6–27). The disease progression occurred in 35 patients (58%). Patients with higher mRNA expression of TLR9 had significantly longer PFS in comparison to patients with lower mRNA expression of TLR9 (*p* < 0.05). The results are shown in Fig. 1.

### TLRs Expression and Infections

Ten patients (17%) suffered from severe infections. Five patients had pneumonia and five patients had infectious diarrhea. Grade 4 infections appeared in patients with advanced disease. In these cases, TLR4 gene expression was decreased in comparison to patients with no or lower grade infections (ΔCt TLR4 4.26 ± 0.62 vs 10.15 ± 2.54). This result was not confirmed by statistical analysis.

## Discussion

B-CLL has a variable clinical course. The following prognostic factors are used in the clinical practice: expression of ZAP70 and CD38, cytogenetic abnormalities and absence of mutations in the IGHV genes whose presence allows the identification of the population of patients with B-CLL with a less favorable prognosis (Zenz et al. 2010).

Despite this, prognostic markers that influence the course of B-CLL are still being searched for. Research has shown that TLRs are actively involved in the regulation of the differentiation and proliferation processes of B cells. In patients with B-CLL, they have shown a decreased gene expression of TLR2 and TLR4 and an increased gene expression of TLR7, TLR9 and TLR10 (Grandjenette et al. 2007; Muzio et al. 2009; Spaner et al. 2006). Previously, Barcellini et al. (2014) assessed the expression of the TLR4 and TLR9 genes in 95 patients with B-CLL. It was found that the expression of TLR4 is lower in the patient population than in the control groups. TLR9 gene expression was higher in patients than in healthy volunteers. Patients with B-CLL and with a decreased expression of TLR4 showed an increased risk of the disease progression and a higher incidence of autoimmune complications (Barcellini et al. 2014). The TLRs expression of genes in patients with B-CLL was also analyzed by Rozková et al. (2010), who demonstrated a reduced gene expression of TLR-4 in patients with B-CLL. Gene expression figures for TLR1, TLR2, TLR6, TLR7 and TLR9 were similar to the control group (Rozková et al. 2010). Arvaniti et al. (2011) marked the TLRs gene expression in a population of 192 patients with B-CLL. Their research confirmed a higher expression of TLR7 and a decreased expression of TLR2 and TLR4 in patients with B-CLL (Arvaniti et al. 2011).

Our research on a population of 60 patients with B-CLL has demonstrated a decreased expression of the TLR4 gene compared with the healthy control group. TLR4 expression was also reduced in patients with advanced stages of B-CLL, as well as in patients with the expression of ZAP-70 and CD38, which are unfavorable prognostic factors in B-CLL. Our observations on the reduced expression of the TLR4 gene are analogous to the data available in the literature and show an influence of TLR4 on the clinical course of B-CLL. By analyzing the gene expression of TLR2, we have demonstrated a higher gene expression of TLR2 in the advanced stages of B-CLL. In the case of the TLR9 gene expression, our findings have proved inconsistent with the results presented in other studies. We have observed that the expression of the TLR9 gene was significantly higher in healthy subjects than in patients with B-CLL. The result of the impact of the TLR9 gene expression on PFS in the studied group is also interesting. In patients with B-CLL and higher, TLR9 gene expression correlated with significantly longer time relapsed before disease progression. This observation, however, requires confirmation in a larger group of patients with B-CLL. In patients with B-CLL, complications after serious infections often occur in connection with the impaired immune function of B cells. In the analyzed group of patients who experienced clinically significant infections, the TLR4 gene expression was reduced which may indicate the participation of TLRs in immune abnormalities in B-CLL.

Our study shows a significant influence of TLR expression, and particularly of TLR4, on the clinical course of B-CLL. The decreased expression of TLR4 gene may suggest an unfavorable prognosis in patients with B-CLL. This observation, however, is preliminary and requires further research in a larger population of patients with B-CLL.
